# Phylogenomic Perspective on the Relationships and Evolutionary History of the Major Otocephalan Lineages

**DOI:** 10.1038/s41598-017-18432-5

**Published:** 2018-01-09

**Authors:** Wei Dai, Ming Zou, Liandong Yang, Kang Du, Weitao Chen, Yanjun Shen, Richard L. Mayden, Shunping He

**Affiliations:** 1 Key Laboratory of Aquatic Biodiversity and Conservation of Chinese Academy of Sciences, Institute of Hydrobiology, Chinese Academy of Sciences, Wuhan, Hubei 430072 People’s Republic of China; 2 University of Chinese Academy of Sciences, Beijing, 100039 People’s Republic of China; 3 College of Fisheries, Huazhong Agricultural University, Wuhan, 430070 People’s Republic of China; 4 Key Laboratory of Freshwater Animal Breeding, Ministry of Agriculture, Beijing, 430070 People’s Republic of China; 5 Department of Biology, Saint Louis University, Saint Louis, MO 63103 USA

## Abstract

The phylogeny of otocephalan fishes is the subject of broad controversy based on morphological and molecular evidence. The primary unresolved issue pertaining to this lineage relates to the origin of Characiphysi, especially the paraphyly of Characiformes. The considerable uncertainty associated with this lineage has precluded a greater understanding of the origin and evolution of the clade. Herein, a phylogenomic approach was applied to resolve this debate. By analyzing 10 sets of transcriptomic data generated in this study and 12 sets of high-throughput data available in public databases, we obtained 1,110 single-copy orthologous genes (935,265 sites for analysis) from 22 actinopterygians, including 14 otocephalan fishes from six orders: Clupeiformes, Gonorynchiformes, Cypriniformes, Siluriformes, Characiformes, and Gymnotiformes. Based on a selection of 125 nuclear genes screened from single-gene maximum likelihood (ML) analyses and sequence bias testing, well-established relationships among Otocephala were reconstructed. We suggested that Gymnotiformes are more closely related to Characiformes than to Siluriformes and Characiformes are possibly paraphyletic. We also estimated that Otocephala originated in the Early-Late Jurassic, which postdates most previous estimations, and hypothesized scenarios of the early historical biogeographies of major otocephalan lineages.

## Introduction

**Figure 1:**
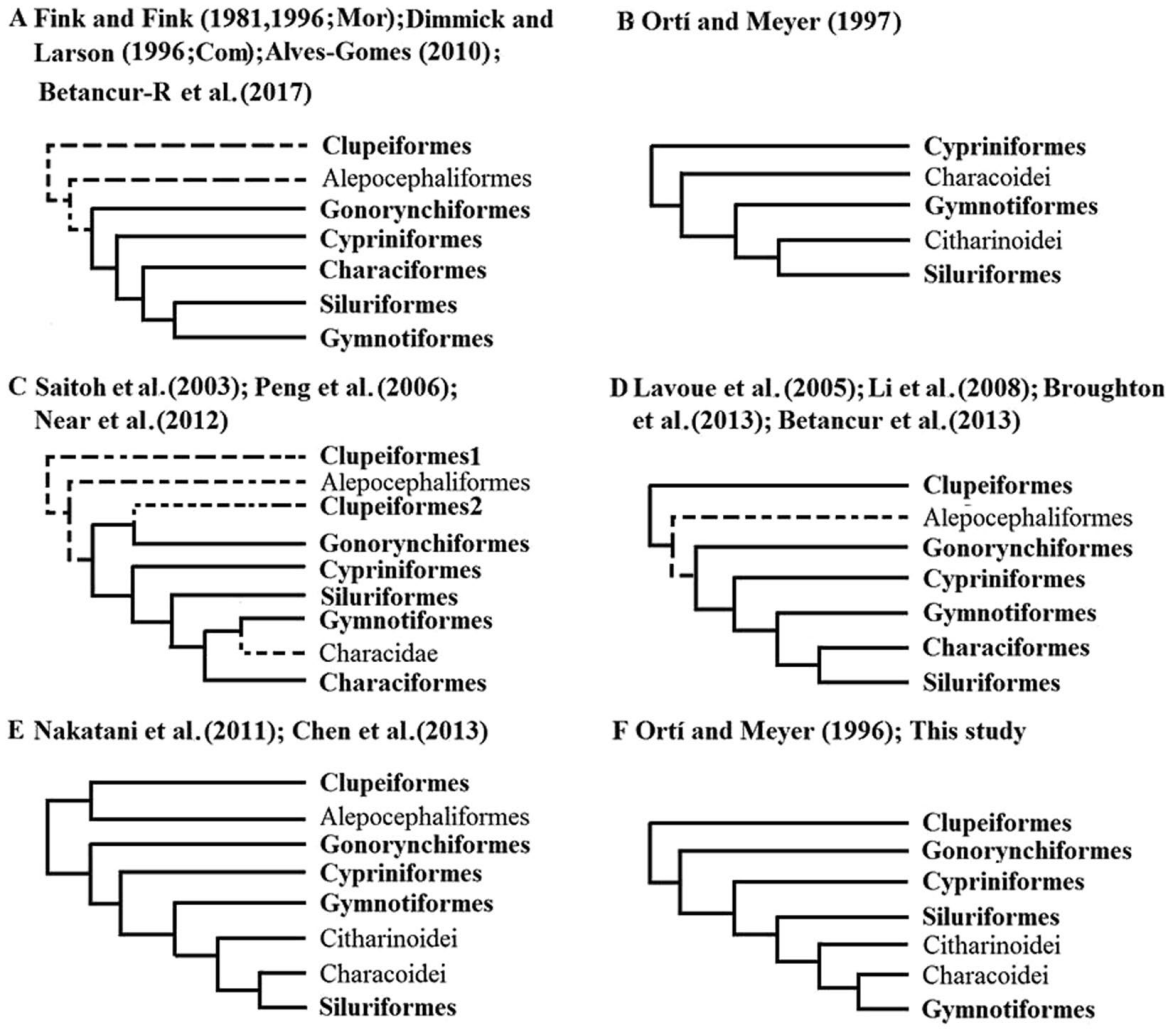
Hypotheses on ordinal relationships of Otocephala through years. (**A**) Fink and Fink^10,12^ (Mor), Dimmick and Larson (Com)^11^, Alves-Gomes^19^ and Betancur-R^59^; (**B**) Ortí and Meyer^14^; (**C**) Saitoh *et al*.^15^, Peng *et al*.^17^ and Near *et al*.^21^; (**D**) Lavoue *et al*.^16^, Li *et al*.^18^ and Broughton *et al*. (2003); (**E**) Nakatani *et al*.^20^ and Chen *et al*.^22^; (**F**) Ortí and Meyer^13^ and this study. ‘Mor’ or ‘Com’ indicates trees were based on only morphological data or combination of morphological and molecular data and others were based on only molecular data. The topology with dotted lines means not all branches included in the studies.

Otocephala has been placed monophyletically as the sister group to Euteleostei^1^. Before Otocephala was defined by Arratia in 1997^2^, the relationships of the major lineages in the clade, based on morphological evidence, have been proposed^3^. A sister relationship of Clupeomorpha and Ostariophysi has been hypothesized since 1995 by Lecointre^4^, which is supported by both morphological and molecular evidences^2,4–9^. A limited number of studies have attempted to resolve the phylogenetic problems within otocephalans^10–22^; however, a number of these studies have called into question the basal relationships of otocephalans with the proposed monophyly Gonorynchiformes and Clupeiformes (Fig. 1C)^15,17,23^ or the monophyly of Clupeiformes and Alepocephaliformes (Fig. 1E)^20,22^. Conflicts are observed in the ordinal relationships among the basal lineage Characiphysi (Fig. 1A,C and D)^10–12,15–19,21,24^. Characiphysi consists of Gymnotiformes, Characiformes and Siluriformes, which together were identified as the sister group to Cypriniformes by Fink and Fink^10,12^. In particular, the monophyly of Characiformes has aroused broad controversy over the last two decades, and molecular-based studies have suggested that Characiformes may be paraphyletic with the recognition of Characoidei and Citharinoidei (Fig. 1B,E and F)^13,14,20,22^.

Uncertainty of relationships and in some cases unresolved relationships have hindered the identification of an accurate time-calibrated origin and biogeographic pattern of the clade because of its worldwide distribution and remarkable species diversity^25,26^. Over the past decade, the primary methods for inferring divergence times of otocephalans has been the identification of characters derived from molecular and fossil materials^15,17,19–22,27,28^. As discussed by Arratia^2^, the earliest occurrence of crown otocephalans was †*Tischlingerichthys viohli*, which has been dated to approximately 150.8–149.8 Mya (see Calibration B in Supplementary Text); however, the actual age of the clade is uncertain^19,20,22,29,30^. Results of studies that have done the time-calibrated trees vary widely^17,20–22,27,31^. The latest published age estimate for the origin of otocephalans is the Early-Late Jurassic^22,27^, whereas the earliest estimate is the Early Permian to the Early Triassic^17,20,31^ (see Supplementary Table 1). Discordance in different studies has resulted largely from the various categories and sizes of selected molecular markers^32–35^, the application of different taxonomic scales and the dating of internal nodes^20,22^. Discrepancies arising from this uncertainty of time estimation have resulted in discrepant hypotheses on the evolutionary patterns of otocephalans because speciation within it has been closely related to geological events occurring at different ages. For example, whether the Characiphysi clade diverged earlier or later than the complete separation of South America and Africa is contentious, and the answer to this question has always been critical to understanding the present geographic distribution of the whole group under tectonic movements and subsequent vicariant events, especially for the strictly South American Gymnotiformes and with respect to the distant relationship of the Neotropical and African Characiformes^20–22,29,36–39^.

Increased taxon sampling relative to the nodes of interest was beneficial to resolving phylogenetic problems^40–43^. Nonetheless, utilizing characters with appropriate evolutionary rates can be more sensitive for yielding robust phylogenetic confidence than the use of additional taxa^35^. Further, acquiring a sufficient number of highly conserved loci may lead to a more accurate site-rate estimation^44^ because the loss of historical signals and systematic bias can be decreased^45–49^, even if the number of analyzed taxa is constrained. Concatenations of fewer than twenty genes have been shown to result in good support for the branch favoring the incorrect topology in yeast phylogenetics^45^. In a simulation analysis of eukaryote phylogeny, several nodes could only be resolved using a phylogenomic approach^50^. Accordingly, phylogenomics appears to be a reliable resolution method, providing an opportunity to generate high-throughput data by capturing expressed sequence tags (ESTs). This work benefited from impressive advances in next-generation sequencing (NGS) technology, which has been broadly applied to resolve phylogenies across diversified taxa but otocephalans^46,49,51–54^.

Herein, 10 novel sets of transcriptomic data were generated via the application of 12 sets of high-throughput data available on public data platforms. To locate orthologous clusters, we created “one-to-one” core-ortholog sets from 8 sets of well-characterized genome data. A total of 1,110 single-copy orthologous nuclear genes with 935,265 positions were obtained based on these core-ortholog sets for phylogenetic analyses. By analyzing each single-gene maximum likelihood (ML) tree, 129 orthologous alignments were screened for bias detection. Then, a well-resolved and robust phylogeny was constructed from a concatenation of 125 bias-excluded ortholog alignments representing 14 otocephalans and 4 outgroup species. We applied a relaxed-molecular-clock analysis to estimate divergence times in 18 taxa of Otocephala based on seven fossil-based calibrations. Finally, using resolved relationships the historical biogeography of the major otocephalan lineages was examined.

## Results

### Data Summary for 18 Species

The number of orthologous genes screened from species varied from 9,619 (*Chanos chanos*) to 25,550 (*Danio rerio*). The supermatrix of 1,100 orthologous genes represented a total of 13,654,221 bp (4,551,407 amino acids), with a loss of 8.6% (see Supplementary Figs S1–S4 and Supplementary Table 2). The contrast in the length distribution of the orthologous genes before and after trimming among the 14 species is shown in Supplementary Fig. S5 (another orthologous genes of 4 species were from 8-species-genome COGs). Sequences for all 28,436 positions in the 22 species were evenly distributed except for two continuously unmapped areas in *Osteoglossum bicirrhosum* and *Myxocyprinus asiaticus* (see Supplementary Fig. S6). We obtained 129 genes by examining relationships among the lineages of each tree inferred from the 1,110 genes. Based on the bias detection, less than 20 out of 1110 genes appeared to provide heterogeneous signals and affirmed that the 129-gene dataset is appropriate for a phylogenetic analysis (see Supplementary Fig. S7 and Supplementary Table 3).

### Best Topology Inferred from the 125-gene Dataset

**Figure 2:**
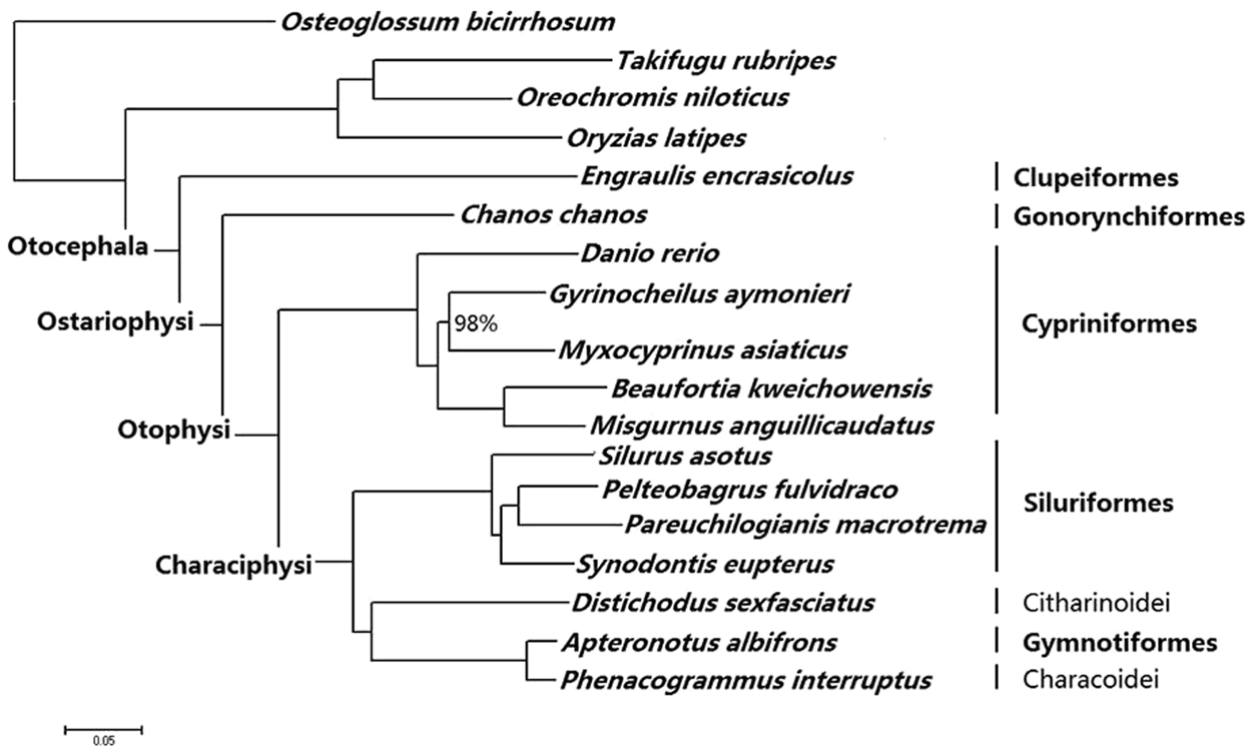
The best-scoring maximum-likelihood tree of Otocephala based on 125 genes (152,223 positions) derived from the bias detection (standard deviation of the tip-to-root distances) on the 1110-genes nuclear matrix with GTRGAMMA model implemented in RAxML. The tree is rooted with *Osteoglossum bicirrhosum*. All nodes with BS = 100% except where noted to be below 100%.

Nine different topologies were created from eight datasets with P-values ≥ 0.05 for the five candidate topologies under the approximately unbiased (AU) test (see Supplementary Table 4). P-values of 1 were obtained for all tests of the topology generated from the 125-gene matrix (152,223 positions) based on the standard deviation (SD) detection of the 1,110-gene matrix (4 genes with Long Branch (LB) attraction or heterogeneity were excluded by calculating the SD of the tip-to-root distances); this was regarded as the best topology in this study (Fig. 2). In the best topology, except for the node Gyrinocheilidae-Catostomidae, which had bootstrap replicate scores (BS) of 98%, all of the nodes were fully supported with BS values of 100%. In addition, consistency was observed as to the best topology using three other datasets that were separately generated from the 129 single-copy gene dataset after detecting the average of the upper quartile (AUQ), and the slope (SL) and R^2^ fit (R^2^) of the linear regression of patristic distances (PDs) against uncorrected distances ‘p’. All the nodes of topologies created from the three datasets also had high support.

Incongruence was mainly concentrated in Characiphysi, although the sister relationship of Characoidei and Gymnotiformes was strongly supported in all of the topologies. The extremely short branches for the Gymnotiformes-Characoidei clade across all of the candidate topologies indicate that Gymnotiformes is more related to Characiformes than to Siluriformes. Siluriformes rooted in Characiphysi had the same support as another candidate topology derived from the ML analysis from the 1,110-gene protein matrix (28,067 positions), with BS = 100% support in the best topology (see Supplementary Fig. S11). However, the Characoidei-Gymnotiformes clade rooted in Characiphysi was fully supported in two other candidate topologies (see Supplementary Figs S8 and S10), whereas Citharinoidei rooted in Characiphysi was supported in the last candidate topology (see Supplementary Fig. S9). In the best topology, Citharinoidei was placed as the sister group of the Gymnotiformes-Characoidei clade, which is consistent with the candidate topology derived from the protein matrix with 28,067 positions (see Supplementary Fig. S11). The Gymnotiformes-Characoidei clade was not supported as a sister group to Siluriformes or to Citharinoidei in the candidate topologies (Supplementary Figs S9 and S10). However, the Citharinoiedei-Siluriformes clade presented BS = 53% for one candidate topology and BS = 84% for another (see Supplementary Figs S8 and S10).

For the major otocephalan lineages, our results support the topology (Clupeiformes, (((Cypriniformes), (Siluriformes, ((Characoidei + Gymnotiformes), Citharinoidei))), Gonorynchiformes)). In addition, the inner relationships among Cypriniformes were inferred as (Cyprinidae + ((Catostomidae + Gyrinocheilidae) + (Gastromyzontidae + Cobitidae)). These relationships were recovered by two candidate topologies, each of which had BS > 95% from the concatenated nuclear matrix (84,201 bp) and the protein matrix (28,067 aa) without gaps (Supplementary Figs S8 and S11). This finding is congruent with the phylogeny of Saitoh *et al*.^55^, which was inferred from whole mitochondrial genome sequences (14,563 bp) of 53 species of Cypriniformes. The best supported topology (Siluridae + (Mochokidae + (Sisoridae + Bagridae))) among Siluriformes was congruent with the three candidate topologies with BS > 75% for each node from the concatenated nuclear matrix without gaps and with half gaps (84,201 bp and 935,265 bp, respectively) and the protein matrix without gaps (28,067 aa) (Supplementary Figs S8–S10).

### Time Estimation Reveals Late Pangaea Origin of Otocephala

**Figure 3:**
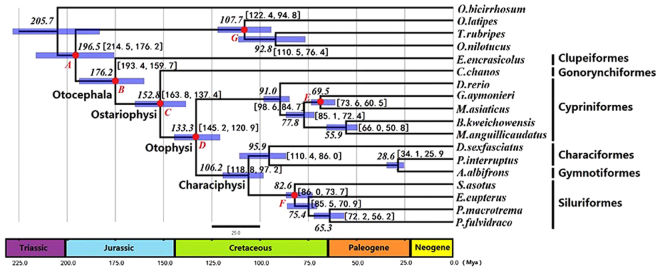
Time-calibrated phylogeny of major otocephalan lineages using BEAST from 90 million generations and seven fossil constraint ages based on the best-scoring maximum-likelihood tree. Numbers on the nodes were the estimated age for the clade. Bars represented the range of 95% high posterior density with the numerical range in square brackets. Red solid round indicated the fossil records used in this study with (**A–G**) corresponding fossil calibration A–G in Supplementary Text. The tree was scaled to the absolute geological time scale in millions of years.

The phylogenetic resolution of Otocephala based on the 125 concatenated nuclear markers offered the basis for inferring their divergence time. A molecular clock analysis was implemented to estimate the divergence time of Otocephala through 125 concatenated nuclear genes using Beast v1.8.3^56,57^. The fossil age constraints are primarily based on Benton *et al*.^28^, who has performed the latest work on the fossil records of animals (see Supplementary Text). Results of the divergence time estimation for Otocephala using 18 species under an uncorrelated relaxed-clock model (see Fig. 3) implied that the age of otocephalan fishes was 176.2 Mya (95% high posterior density [HPD]: 193.4–159.7 Mya) in the Toarcian age of the Early Jurassic. This finding is consistent with the age deduced from the most basal Ostariophysan fossil †*Tischlingerichthys viohli* (228.4–149.8 Mya; see Calibration B in the Supplementary Text). Generally, our divergence offers a time interval that is compatible with all of the minimum ages and most of the soft maximum ages provided by seven fossil records (see Fig. 2 and Supplementary Text). Our results are conservative compared with those of other studies, which present age estimations for almost all lineages that pre-date ours. Our estimate is far younger than the estimations of Peng *et al*.^17^ (279 Mya, HPD: 293–264 Mya) and Nakatani *et al*.^20^ (265 Mya, 286–243 Mya) but similar to that of Chen *et al*.^22^ (177 Mya) and only slightly earlier than that of Santini *et al*.^27^, who estimated 151 Mya for the origin of the clade (see Supplementary Table 1).

Based on the above results, the inferred age of the ostariophysan lineage is 152.8 Mya (HPD: 163.8–137.4 Mya) in the Kimmeridgian age of the Late Jurassic; that of the otophysan fishes is 133.3 Mya (HPD: 145.2–120.9 Mya) in the Hauterivian age of the Early Cretaceous. Both of these inferred ages are within the range of fossil ages (see Calibrations C and D in the Supplementary Text). The estimated divergence age of characiphysan fishes ranged from 118.8 to 97.2 Mya, which corresponds to the Albian age of the Early Cretaceous. We estimated a Cretaceous origin of extant Cypriniformes between 98.6 and 84.7 Mya, which is consistent with the fossil age constraints (see Calibration E in Supplementary Text), as well as the Siluriformes clade between 86.0 and 73.7 Mya, which is also compatible with the fossil age (see Calibration F in Supplementary Text). The inferred time of divergence for Characoidei and Gymnotiformes was 28.6 Mya (HPD: 34.1–25.9 Mya) in the Rupelian age of the Oligocene.

## Discussion

This study applied bias detection to high-throughput data, and with this novel process has yielded the greatest amount of information thus far for Otocephala; moreover, this method was also able to resolve the phylogeny of major otocephalan lineages and represents a heuristic approach to fish phylogenomics. High-throughput analyses that combine genomic and transcriptomic data can balance the taxa and characters required to infer phylogenetic relationships because a sufficient number of historical signals could be obtained by using an optimal proportion of these data sources. The robust relationships among the major lineages pass repeated tests and offer a novel perspective on the historical biogeography of the lineages.

Though the major lineages (incertae sedis notwithstanding) of Otophysi have been grouped together morphologically since 1911 by Regan^3^, their relationships were still controversial^10–22^. Our analyses strongly support Gonorynchiformes as the basal group of ostariophysans. However, Gonorynchiformes and Clupeiformes were grouped together in the phylogenetic analysis of Ishiguro *et al*.^15,17,23^. This grouping was likely because of LB attraction, also explain the results obtained by Saitoh *et al*.^15^ and Peng *et al*.^17^ despite their use of more characters. In this section, we focused on the Characiphysi clade, which is an assemblage that has attracted broad controversy.

Prior to the definition of Otocephala, Siluriformes was considered the basal group of Otophysi^3,58^; however, this was never recovered in subsequent studies. Using 127 characters, Fink and Fink^10^ hypothesized that Gymnotiformes formed the sister group of Siluriformes, and Gymnotiformes plus Siluriformes was sister to Characiformes; this hypothesis is also emphasized in their updated work^12^ in 1996 and other molecular-based hypotheses^11,19,59^ (Fig. 1A). If this assertion is true, then one overriding question relates to when and where the common ancestor of Gymnotiformes and Siluriformes arose. Moreover, why is Gymnotiformes endemic to the Neotropics while Siluriformes occupies almost all continents? Alternative scenarios are difficult to propose based on the hypothesis of Fink and Fink^10^.

In some other studies, Siluriformes formed the sister group to Characiformes, and together the clade formed the sister group to Gymnotiformes^16,18,24,36^ (Fig. 1D). Nevertheless, the prevalent hypothesis supports a divergent relationship with Siluriformes as the sister group to the Gymnotiformes plus Characiformes clade^15,17,21^ (Fig. 1C). This assertion is not surprising as the notion has been proposed even before Fink and Fink^3,58^. Interestingly, Mago-Leccia and Zaret^60^ performed anatomical and ecological analyses and found several common morphological characteristics among Gymnotiformes and Characiformes. However, if this association is correct, then why the electroreceptive system only appears in Siluriformes and Gymnotiformes among Otophysi remains unresolved.

Dimmick and Larson^11^ speculated that parallel informative substitutions on a very short lineage of Gymnotiformes and Siluriformes were transcended by those on a long lineage evolving to Gymnotiformes and Characiformes. Deep within the phylogenetic tree, functional characters on short branches were genetically fixed and more likely to be recovered morphologically. Alternatively, the electroreceptive system may have originated twice: first during the divergence of Siluriformes and later during the divergence of Gymnotiformes. This independent origin of electroreception echoes the hypothesis of Chen *et al*.^22^ where in it was hypothesized that the common ancestor of Siluriformes and Gymnotiformes was electroreceptive^10,19^.

The nonmonophyly of Characiformes was first proposed in 1996^13^. In addition to Ortí and Meyer^13,14^ (Fig. 1B and F), two other studies have questioned the monophyly of Characiformes^20,22^ (Fig. 1E). Most molecular-based phylogenies of Otophysi that have characterized the order as monophylic included no more than two representatives of Characoidei^15–18,36^. Chen *et al*.^22^ even reanalyzed the dataset of Dimmick and Larson and found that Characiformes was paraphyletic with respect to Gymnotiformes^11,22^. Interestingly, although there was no any Citharinoidei as sample, Characiform nonmonophyly was still obtained by Peng *et al*., who indicated that Characidae was closer to Gymnotiformes than to Alestidae^17^. Our hypothesis of Otocephala is almost coincident with that of Ortí and Meyer in 1996^13^ (Fig. 1F), who examined relationships of 25 teleost fishes using alignments of Ependymin. Their analysis suggested Distichodontidae was the sister group of Gymnotiformes and Characoidei only when transitions in the third positions were excluded. Notably, Alestidae was always grouped into Neotropical Characiformes, although the monophyly of Alestidae was never supported under hypotheses of alternative weighting strategies. Similar to this study, the inner branch of Alestidae deep within the phylogenetic tree was as short as that of Gymnotiformes and only included *Eigenmania* and *Rhamphichthys* instead of *Apteronotus*. If the hypothesis of Ortí and Meyer is reliable, then the origin of Gymnotiformes might be earlier than current estimates because Gymnotidae was generally recognized as the basal group of all remaining Gymnotiformes^61,62^. However, based on the electrical potential of *Electrophorus*, Alves-Gomes implied that gymnotiform electric eels might have evolved faster than other clades in Otophysi^19^.

Gymnotiformes were hypothesize as a “specialized^63^” or “highly modified^60^” group within Characiformes in some research. As for Citharinoidei, in summarizing several plesiomorphic features Fink and Fink^10^ suggested that Citharinidae and Distichodontidae formed a monophyletic group and represented the most ancient of Characiformes. Interestingly, this hypothesis is supported by most molecular-based studies^13,19–22^, including ours, but not by studies of Ortí and Meyer^14^. However, our results support that Characoidei are more closely related to Gymnotiformes than to Citharinoidei.

We asserted the origin of Otocephala in the Toarcian age of the Early Jurassic when Gondwana began to rift between North America and Africa in the Early-Middle Jurassic (~175 Mya) (see Fig. 3). The separation of Africa and South America is broadly accepted to have involved multiple geological events that occurred over a period of more than 100 My and included a series of vicariant-dispersal events^64,65^. Consistent with the hypotheses proposed by Chen *et al*.^22^, our hypotheses were not fully supported by the scenario in which a portion of the dispersal of Characoidei and Siluroidei occurred sooner than or as a result of the separation between Africa and South America as proposed by Lundberg^29^ and Briggs^30^. Because both suborders appeared so late, based on our inference, Africa and northeast Brazil may have remained connected before the end of the Cretaceous^66^. The scenario of otocephalan biogeography is hypothesized as follows.

I. The fossil †*Tischlingerichthys viohli*, formed by soft carbonate muds from the bottom of lagoons in the Mörnsheim Formation, was found in southern Germany (Mühlheim, Bavaria)^67^. Thus, our scenario implies that the otocephalan ancestor inhabited a marine environment in the eastern part of the Tethys Ocean in the Early Jurassic approximately 176 Mya, when Pangaea was rifting. This period experienced swift geological change because of the resulting formation of oceans and tropical climate over the formerly dry region in the Pangaea^20^.
II. Because fossil Gonorynchiformes have been found in marine deposits located in Germany^68,69^, Spain, and Italy^70^ close to the original areas that split the two major land masses, Laurasia and Gondwana, we infer that Ostariophysi might have originated in the Eurasian offshore ocean approximately in the Late Jurassic before the final separation of South America and Africa. Furthermore, the living genera of Gonorynchiformes appeared to have had a saltwater life similar to other basal teleosts, such as *Albula* and *Elops* ^10,19,70^.
III. Occurring in marine (e.g., Chanoides^71^) or brackish waters (e.g., *Santanichthys* ^72,73^), the original otophysans split into two groups roughly in the Early Cretaceous that became the extant Cypriniformes and Characiphysi. The most ancient otophysan fossil, †*Santanichthys diasii*, was found in approximately the Early-Late Cretaceous, implying a Gondwana origin of the common ancestor of otophysans. Although our estimation of the age of otophysans postdates the final separation of South America and Africa, the last land bridge between the two plates remained until ~102 Mya^19,74^, implying potential opportunities for these species to colonize the neighboring continent.
IV. With over 3,500 species, Cypriniformes has been argued to be the most diverse order of freshwater fishes^75^. As Alves-Gomes^19^ speculated, the fauna of otophysans occupying Asia formed the common ancestor of Cypriniformes, inferred from the tremendous diversity of Cypriniformes in China^25,26^. This finding is consistent with the inference of Saitoh *et al*.^31^, which was used to date the basal cypriniform divergence to 155.9 Mya. According to our time-calibrated phylogeny, the differentiation of Cypriniformes occurred approximately 98.6–84.7 Mya in the Turonian age of the Cretaceous and was probably promoted by the strong orogenies in the Late Cretaceous, which accelerated speciation. The fossil age of the Catostomidae and Gyrinocheilidae clades was estimated at 73.6–60.5 Mya, an estimate that could explain the distribution of Catostomidae and Cyprinidae in North America by assuming that Greenland and Labrador formed the pathway for dispersal; North America and Europe were still connected until 49–47 Mya^19^.
V. The migration of Africa and South America at approximately 100–95 Mya represented a vicariant event dated speciation of the ancestral Characiformes to approximately 110.4–86.0 Mya based on our estimation. The age estimation was also consistent with the age inferred from †*Santanichthys diasii*, which was considered the most ancient Characiformes. Because the age estimation of Characiphysi was approximately 100 Mya, the dispersal of the ancestor of the freshwater lineage Characiphysi was assumed to be accelerated by a major marine transgression in the Late Cretaceous that isolated the western part of North America from the remaining Pangaea with an epicontinental sea^19^. More basal Characiformes also appeared during this period at about 119–68 Mya, and they likely covered South America and Africa based on the location of †*Santanichthys diasii*, which was located in Brazil, as well as on the present distribution of Citharinoidei.
VI. The estimated age of Siluriformes was about 86–74 Mya, and the oldest Siluriformes fossils were from Campanian (84–74.5 Mya) deposits in South America^29^. In addition, marine Siluriformes fossils were found in Late Cretaceous deposits of North America and Eocene deposits of southeastern Arkansas^19,29^. Molecular evidence confirmed that the clade in South America were the earliest Siluriformes^76–79^; if this is true, then the worldwide distribution of this group could only have occurred via one pathway under our scenario. Marine transgression permitted this group to move to other continents as suggested by Roberts^63^, and this hypothesis could also explain the tolerance to salt water of current Siluriformes clades, such as Aspredinidae, Auchenipteridae, Arridae, and Plotosidae^19,63^. Furthermore, the available paleogeographical and paleoecological data support the presence of a land bridge between Brazil and Africa until the end of the Maastrichtian (66 Mya) in the Late Cretaceous. This bridge would have offered narrow faunal links for the exchange of planktonic foraminifera and other species^66^. However, a monophyletic Siluriformes is not represented in both the South American and African lineages as previously reported^19,29,76,77^.
VII. The differentiation between Gymnotiformes and Alestidae occurred approximately 29 Mya, which surprisingly postdates the ages estimated in previous studies^15,17,20–22^. Because our study was restricted to particular taxa, we are unable to discuss the subgroup of endemic to South American Characiformes. However, following the phylogeny of Triporthidae proposed by Mariguela *et al*.^79^, the estimated age of Characidae in central and South America was 42.3 ± 12.9 Mya based on the fossil constraint age of †*Lignobrycon ligniticus* (28.5 ± 5.5 Mya)^79,80^. This dating is compatible with our inferences because the origin of Neotropical Characiformes definitely occurred earlier than that of the African lineage. If our estimation is correct, then an alternative explanation may be available for the scenario in which the common ancestor of Gymnotiformes/African Characiformes was isolated in Neotropics for a period forming Neotropical Characiformes. Likely close to the same time, along with the largest marine transgression in the Early Cenozoic, a partial fauna belonging to the common ancestor of Gymnotiformes/African Characiformes (probably including the common ancestor of Alestidae) arrived in Africa via transcontinental connections as the basal African Characiformes. Gymnotiformes arose as a portion of this clade in south-central South America.

The crustal tectonism that frequently occurred in the Cenozoic of South America subsequently permitted Gymnotiformes to move into other Neotropical areas, including the Amazon Basin. As Saitoh *et al*.^15^ hypothesized, Gymnotiformes also arrived in Africa, failed to compete with Mormyridae, which used a similar ecology of electrolocation, and became extinct. This hypothesis was based on the following findings: (i) most characiform subgroups endemic to the Neotropics were not closely related to groups in Africa^29,39,81^; (ii) extant Citharinoidei were endemic to Africa, whereas Gymnotiformes were strictly endemic to South America and southern Central America; and (iii) the only well documented gymnotiform fossils were specifically from the Yecua formations in Bolivia, which were dated to about 11–10 Mya^82,83^. Fossils of Gymnotiformes from this early time period (as inferred in certain studies) are rare. Moreover, because the fossil taxa of otophysans originated in marine or brackish water, we could not deny the salinity tolerance of the common ancestor of Gymnotiformes/African Characiformes despite the freshwater restraint of extant Characiformes^65,84^. As discussed by Ortí and Meyer, Citharinoidei were likely not related to Alestidae, and molecular and morphological evidence suggested that two levels of the African and South American subgroup occurred, with one formed by Distichodontidae and the remaining Characiformes and the other formed by Alestidae and the South America subgroup^13,85,86^. Furthermore, our assumption regarding the approximate relationship of South American and African Characiformes does not conflict with the hypothesis of Calcagnotto *et al*.^65^. The sister group of Citharinoidei, Characoidei, is composed of two lineages: one represented a clade of both African and Neotropical taxa, and the other included African Alestidae sister to two Neotropical families and the African Hepsetidae. This assemblage was sister to two other Neotropical families. The other lineage was a strictly Neotropical clade consisted of the remaining Characoidei. Ortí and Meyer^13^ suggested that Alestidae are Neotropical Characiformes, which implies that Alestidae were early visitors to Africa and were derived from the common ancestor of Gymnotiformes/African Characiformes under our scenario.

## Materials and Methods

### Taxon Sampling and Data Collection

We collected 10 commercial species representing 9 genera of 5 orders in Otocephala and 1 genus of Osteoglossiformes as the root. We then used Illumina paired-end RNA sequencing technology to create ten new transcriptomic datasets for the following Osteoglossocephalai fishes: one Gonorynchiformes species (*Chanos chanos*), four Cypriniformes species (*Gyrinocheilus aymonieri*, *Myxocyprinus asiaticus*, *Beaufortia kweichowensis* and *Misgurnus anguillicaudatus*), two Characiformes species (*Phenacogrammus interruptus*, *Distichodus sexfasciatus*), one Gymnotiformes species (*Apteronotus albifrons*), one Siluriformes species (*Synodontis eupterus*) and one Osteoglossiformes species (*Osteoglossum bicirrhosum*). Gonorynchiformes and Gymnotiformes were represented by only one species because of sampling difficulties. The raw reads of the 10 species were deposited with the National Center for Biotechnology Information (NCBI) Sequence Read Archive (SRA).

The raw data of Clupeiformes were obtained from external sources; genomic data of *Engraulis encrasicolus* (Clupeomorpha) were retrieved from http://www.ncbi.nlm.nih.gov/sra/SRX315003[accn] (last accessed December 23, 2013). Another 3 transcriptomic data of Siluriformes were obtained from NCBI, including *Silurus asotus* (https://www.ncbi.nlm.nih.gov/sra/SRR1994457/), *Pelteobagrus fulvidraco* (https://www.ncbi.nlm.nih.gov/sra/SRR1994459) and *Pareuchiloglanis macrotrema* (https://www.ncbi.nlm.nih.gov/sra/SRR1994404) (last accessed March 23, 2016). The genomic sequences and the one-to-one orthologous relationships of eight model fish species, *Danio rerio*, *Takifugu rubripes*, *Oryzias latipes*, *Oreochromis niloticus*, *Xiphophorus maculates*, *Gasterosteus aculeaus*, *Tetraodon nigrovirids*, and *Gadus morhua*, were retrieved from http://www.ensembl.org/info/data/ftp/index.html (last accessed December 23, 2013).

### Laboratory Protocols and Data Processing

For each live species, liver tissues of 3–5 individuals were extracted, immediately immersed in RNAlater (Life Technologies, Carlsbad, CA, USA), and frozen on liquid nitrogen until assay. RNAiso Plus reagent (Takara Biotechnology, Dalian, China) was used to isolate total RNA following recommendations of the manufacturer. The crude extract was purified using an RNeasy mini kit (Qiagen, Valencia, CA, USA) to exclude genomic DNA, and a Bioanalyzer 2100 (Agilent) was used to determine the integrity of the sample. The RNA-seq libraries were constructed using the Illumina Gene Expression Preparation Kit (Illumina, San Diego, CA, USA). Briefly, the mRNA was enriched from total RNA using Magnetic Oligo (dT) Beads (Illumina) and fragmented into pieces using the RNA fragmentation kit (Ambion, Austin, TX, USA). Reverse transcriptase (Invitrogen) and random hexamer-primers were used to synthesize the first-strand cDNA, and then DNA polymerase I (NEB) and RNaseH (Invitrogen) were used to synthesize the second-strand cDNA. The double-stranded cDNA was end-repaired by T4 DNA polymerase (NEB), Klenow enzyme (NEB) and T4 polynucleotide kinase (NEB). A single A-base addition was used to prepare the DNA fragments for ligation to the adapters using DNA ligase (NEB). Suitable fragments were selected using a Gel Extraction Kit (Qiagen) and amplified by PCR. These purified products represented the designated cDNA library. The library was paired-end sequenced on an Illumina HiSeq^TM^ 2500 platform.

Low-quality sequences with ambiguous ‘N’ bases and known adapters were filtered to remove reads in which more than 10% of the bases had Q-values < 20. Sequences shorter than 60 bp as well as rRNA sequences that aligned with the SILVA database were discarded to avoid sequencing artifacts. Trinity^87^ was then used to separately assemble the left reads into the resulting contigs for each sample, and the contigs were joined into transcripts. Transcripts longer than 200 bp were selected to construct the sample contig set for further analysis. The SRA data for *Engraulis encrasicolus* were converted into FASTQ data using SRA Toolkit (http://www.ncbi.nlm.nih.gov/Traces/sra/sra.cgi?cmd=show&f=software&m=software&s=software) and processed following the standard protocol as above.

### Core-ortholog Set Generation and Orthology Assignment

In efforts to obtain phylogenetic inferences that would not be affected by misleading history signals, such as ‘hidden paralogs’, we generated 8-species-genome COGs (core-ortholog groups) that were used to search potential orthologs instead of the universal InParanoid database. The HaMStR pipeline^88^ was performed for orthology assignment. Fish-specific genome duplication in teleosts, which may result in “one-to-two” or “one-to-many” rather than “one-to-one” orthology relationships, were considered, and the amino acid sequences of eight model fish species and the corresponding “one-to-one” relationships from Ensembl by BioMart^89,90^ were constructed as the COGs for the putative ortholog search following the procedure for the “Generation of new core-ortholog sets” from the hamstrsearch_local package in HaMStR. We set “5” as the minimum number of sequences for one core-ortholog unit. The sample contig sets of each species were assigned to the COGs using a BLASTX analysis. To acquire similar sequences^91^, BL2SEQ was used to align each translated contig sequence to the best hit from the output of the BLASTX search, and the sequence whose translated format had the lowest E-value was chosen as the optimal candidate. After more than one contig sequence was screened out from the COGs as the ortholog, the shorter sequences were cut off, and then the putative single-copy orthologs were obtained. Using this approach, a total of 1,452 nucleotide and amino acid orthologs among 22 species were extracted from the newly generated COGs representing the most conservative regions (the COGs data on 4 species were excluded from phylogenetical analysis). Each collected locus of the COGs represented an ortholog cluster.

MAFFT v7.222^92^ was used to align each protein ortholog cluster with the parameter “–ep 0, –genafpair, –maxiterate 1,000, –thread 90”, and then PAL2NAL^93^ was applied to align each nucleotide ortholog cluster from the corresponding aligned protein sequences. When mismatches occurred, MACSE^94^ was used to finish the alignment instead. After all of the ambiguous “N” bases were replaced as the gaps, Gblocks^95^ with parameter “-t = c, Allowed Gap Positions = None/with half” were used to trim both ortholog clusters. Ultimately, 1,110 ortholog clusters longer than 60 bp were retained and concatenated to supermatrices by a Python script. To visualize the degree of distribution homogeneity for each locus of each species, a heat-map analysis was created using the R package.

### Inferring Phylogenetic History

To ensure that the optimal outgroup was selected, we performed a ML inference for the protein supermatrices with half gaps of 22 species by running RAxML 7.2.6^96^ for 100 bootstrap replicates under the PROTGAMMAJTTF model. The LB score for each taxon was then calculated using TreSpEx v1.1^97^ based on the ML tree with PDs. By considering the position of the nodes, which were broadly accepted (available at http://www.geocities.jp/ancientfishtree/DivTimeEstimation.html), we retained in the COGs data on 4 species: *Danio rerio*, *Oreochromis niloticus*, *Oryzias latipes* and *Takifugu rubripes*. With the addition of the remaining 14 species screened out from the transcriptome sequences, 18 species used to infer the otocephalan phylogeny included Clupeiformes (1), Gonorynchiformes (1), Gymnotiformes (1), Cypriniformes (5), Siluriformes (4), Characoidei (1), Citharinoidei (1), Osteoglossiformes (1), Perciformes (1), Beloniformes (1) and Tetraodontiformes (1); the latter four orders were used as outgroups. To more clearly illustrate the data, we graphically compared the number of raw reads and mapped reads. Four datasets were finally assembled from both ortholog clusters that represented the nucleotide and protein supermatrices with half gaps and without gaps of 18 species for the phylogenetic analysis. For the nucleotide supermatrices with half gaps, we constructed ML trees of data that were (1) unpartitioned; (2) unpartitioned excluding third codon positions (1,000 bootstrap replicates); (3) partitioned by codon position (designated as 12_n_ + 3_n_, where 1, 2, and 3 represent the 1st, 2nd and 3rd codon positions, respectively, and the subscript “n” represents nucleotides); (4) partitioned by genes; and (5) partitioned by genes excluding 3_n_ under the best-fit GTRGAMMAI model tested by Modeltest^98^ with 100 bootstrap replicates. The ML analysis was also applied to the nucleotide and protein supermatrices without gaps (500 and 1,000 bootstrap replicates, respectively; GTRGAMMAI model) as well as to the protein supermatrices with half gaps (unpartitioned and partitioned by genes, 100 bootstrap replicates; PROTGAMMAJTTF model). Nucleotide supermatrices without gaps were also implemented for a Bayesian Inference (BI) under the GTRGAMMAI model with two independent Monte Carlo Markov chain (MCMC) runs for a total length of 56,000 cycles in PhyloBayes version 4.1^99^. The bpcomp program (maxdiff < 0.1) was then used to determine any discrepancies between the two chains following the burn-in of 5,000 cycles and sub-sampling every 100 trees.

### Regeneration of Extra Datasets with Misleading Signals Excluded

Heterogeneous signals, such as conflicts between genes, LB attraction or saturation of datasets, are known to mislead phylogenetic history reconstructions^97,100–105^. In addition, incorrect phylogenies can be produced with strong support from concatenated genes that share certain biases^106^. Here, TreSpEx v1.1 was also used to detect the LB and saturated partitions of the pruned dataset. First, we implemented the best fit models for 1,110 genes and then performed the ML analysis under the corresponding model for 500 bootstrap replicates. Subsequently, we checked the topology one by one. For each single-gene tree, genes were only retained when the species classified within the same lineage formed one cluster, which allowed *Engraulis encrasicolus* to be grouped together with Euteleostei or *Chanos chanos* by LB attraction. The concatenated dataset from the selected genes minimized the conflict between informative characters. After the average evolutionary rates were calculated as a proxy, the program TreSpEx was used to calculate the AUQ and SD of the tip-to-root distances, which were used as a measurement of LB attraction based on the PDs in the tree^97,101–103,107^. Additionally, the SL and R^2^ of the linear regression of the PDs against the uncorrected distance p for every gene that could be assessed with respect to the degree of saturation were calculated by TreSpEx^97,100,101,108,109^. The density plots of the four indices were then generated with the help of the R package^110^. Genes covered by the sloped and unsmooth section on the right tail of the curve (i.e., high values) followed by an obvious and optimal shoulder were considered to present LB attraction in the detection of either the AUQ or SD; thus, they were excluded. Genes with low values on the left part of the curve were removed because of the apparent high degree of saturation in the detection of either the SL or R^2^. The remaining genes were concatenated for subsequent ML analysis.

Sequence bias detection was executed for the 1,110 gene datasets of 18 species. We obtained 129 genes without bias by examining relationships among the lineages of each tree inferred from the 1,110 genes. Six genes and 4 genes with LB attraction or heterogeneity, respectively, were identified by separately calculating the average of the AUQ and the SD of the tip-to-root distances. Seven genes and 18 genes were separately saturated by the SL and R^2^ of the linear regression of PDs against uncorrected distances ‘p’. Every gene was identified with the aid of TreSpEx, which is considered a useful program for detecting heterogeneous signals such as saturation, LB attraction, paralogy, and conflict between different datasets.

### Conjoint Analysis of Phylogenetic Trees

After determining the AUQ, SD, SL and R^2^, four datasets were generated from the concatenated dataset. We implemented ML analyses for the four datasets with RAxML 7.2.6^96^ under the best fit model for 500 and 1,000 bootstrap replicates. To evaluate the confidence of all topology hypotheses, CONSEL^111^ was used to implement the AU test^112^, the Shimodaira-Hasegawa (WSH) test, the Kishino-Hasegawa (KH) test and the Bootstrap Probability (BP) test after the per site log-likelihoods of each topology were calculated using RAxML 7.2.6 and PAML 4.8^113^. Eight datasets comprised of four 1110-gene datasets that represented the nucleotide and protein supermatrices with half gaps and without gaps and four datasets without bias screened from 129-gene datasets after sequence bias detection.

### Estimation of Divergence Time

Beast v1.8.3^56^ was used to estimate a time-calibrated tree with a node-dating strategy. A BEAST XML file was generated by BEAUTi v1.8.3 using an uncorrelated log-normal-distribution relaxed-clock model and a Yule speciation process as the tree prior. The descriptions of 7 fossil calibrations of the MRCA are presented in the Supplementary Text. The GTR model was used as the substitution model, Gamma + Invariant Sites were used for the site heterogeneity categories, and the Yule tree prior was used for all BEAST runs. As for the prior parameter, ucld.stdev and ucld.mean were set as the uniform distributions. The MCMCs were run in BEAST for 90 million generations with sampling every 1,000 cycles for each dataset. The effective sample sizes of all parameters were > 200. Tracer v1.5 was used to check the stationarity of the MCMC parameter sampling, and TreeAnnotator v1.6.1 (http://beast.bio.ed.ac.uk/TreeAnnotator) was then used to inspect the posterior set of trees, with the first 20% of the sampled trees discarded as burn-in^23^.

### Accession codes

The RNA-Seq data have been submitted to the NCBI Sequence Read Archive (SRA) under the accession numbers SAMN04572094, SAMN04572095, SAMN04572096, SAMN04572097, SAMN04572094, SAMN04572095, SAMN04572096, SAMN04572097, and SAMN04572094.

### Ethical approval

The methods involving animals in this study were conducted in accordance with the Laboratory Animal Management Principles of China. All experimental protocols were approved by the Ethics Committee of the Institute of Hydrobiology, Chinese Academy of Sciences.

## Electronic supplementary material

Supplementary Information
