# Supplementary material for: Phylogenomic Perspective on the Relationships and Evolutionary History of the Major Otocephalan Lineages
Source: Sci Rep. 2018 Jan 9;8:205. doi: 10.1038/s41598-017-18432-5 (PMC5760653; doi:10.1038/s41598-017-18432-5)
Supplement: Supplementary file 1 — Supplementary Information [file 41598_2017_18432_MOESM1_ESM.pdf]

Phylogenomic Perspective on the Relationships and Evolutionary History of the Major Otocephalan Lineages

Wei Dai[1,2]

Email: david86072521@163.com

Ming Zou[3,4]

Email: zoumingr@163.com

Liandong Yang[1,2]

Email: yangliandong1987@163.com

Kang Du[1,2]

Email: dukang@ihb.ac.cn

Weitao Chen[1,2]

Email: chenweitao@ihb.ac.cn

Yanjun Shen[1,2]

Email: shenyanjun@ihb.ac.cn

Richard L. Mayden[5]

Email: cypriniformes@gmail.com

Shunping He[1]*

* Corresponding author

Email: clad@ihb.ac.cn

[1] Key Laboratory of Aquatic Biodiversity and Conservation of Chinese Academy of Sciences, Institute of Hydrobiology, Chinese Academy of Sciences, Wuhan, Hubei 430072, People's Republic of China

[2] University of Chinese Academy of Sciences, Beijing 100039, People's Republic of China

[3] College of Fisheries, Huazhong Agricultural University, Wuhan 430070, People's

Republic of China

[4] Key Laboratory of Freshwater Animal Breeding, Ministry of Agriculture, Beijing 430070, People’s Republic of China

[5] Department of Biology, Saint Louis University, Saint Louis, MO 63103, USA

Supplementary Text. Age priors for the fossil calibration. Each fossil calibration of the most recent common ancestor (MRCA) was mostly based on the latest published estimation, e.g., Benton, et al. (2015)[1]. Seven calibrated nodes were separately listed the taxa (except the calibration E) represented the oldest fossil record for the lineage, the phylogenetic position analyzed from morphological characters and age justification concluded from previous stratigraphy analysis.

Calibration A. Node: Crown lineage Clupeocephala. MRCA: †*Leptolepides haerteisi* from the Solnhofen Formation of Bavaria, Germany[2]. Phylogenetic identification: sister clade of Salmonoidei and Esocoidei represented the age between Jurasic and Cretaceous[2]. Age justification: the upper age of the zone where the fossils found was 150.94 Mya which inferred as the minimum age of the clade[3]. The zones where the oldest stem teleosts fossils found were estimated at the latest Ladinian, derived from which 242 Mya was dated as the upper bound for the crown lineage Clupeocephala[1,4-6].

Calibration B. Node: Crown lineage Otocephala. MRCA: †*Tischlingerichthys viohli* from the Mörnsheim Formation of Mühlheim, Bavaria, Germany[1,2,7]. Phylogenetic identification: the most basal ostariophysan within Gonorynchiformes from the Upper Jurassic (150.8-149.8 Mya) via morphological analysis [1,2,8-10]. Age justification: As for no crown teleosts fossil found, any primitive teleost was evolved during the Carnian now dated about 235 Mya, from which we set 149.8 Mya as the minimum age and 228.4 Mya as the soft maximum age[1,6,11,12].

Calibration C. Node: Crown lineage Ostariophysi. MRCA: †*Rubiesichthys gregalis* from El Montsec lithographic limestone, Lérida, Spain[13,14]. Phylogenetic identification: the oldest Chanidae within crown Gonorynchiformes from the Early Cretaceous (145.5-139 Mya) first described by WENZ in 1984. Age justification: From the deposits of ancient organisms where Rubiesichthys fossilized under the marine limestones, the late Barremian-early Aptian dated as 130.8 to 126.3 Mya was estimated, from which the minimum age of 126.3 Mya set[15-18]. No more older fossil of the stem Chanidae appeared in that age, before when only †*Tischlingerichthys viohli* represented the crown Otocephala from the late Jurassic (Kimmeridgian-Tithonian) dated to 157.3 Mya ± 1.0 Mya, 158.3 thus derived as the maximum age[1,19,20].

Calibration D. Node: Crown lineage Otophysi. MRCA: †*Santanichthys diasii* from the Santana Formation of the Araripe Basin, northeastern Brazil[21,22]. Phylogenetic identification: the oldest Otophysan fossil as a stem Characiform from the early Cretaceous (112-99.6 Mya)[22]. Age justification:

99.6 Mya was used as the minimal age offset and 158.3 Mya as the soft upper bound based on the Kimmeridgian (157.3 ± 1.0 Myr) (see details in node B).

Calibration E. Node: Stem lineage of Gyrinocheilidae + Catostomidae. MRCA: †*Amyzon mentale* from the Osino shales, Nevada, USA[23]. †A*myzon brevipenne* from the Pleasant Valley, British Columbia, Canada. †*Amyzon aggregatum* from the Fontenelle Reservoir, Wyoming, United states[24,25]. Phylogenetic identification: †*Amyzon mentale* was the first Catostomid fossil in Cypriniformes from the Early Paleocene (65.5-61.1 Mya) and the later two were closely correlated to the group including Ictiobus and Carpiodes from about 49.4 Mya[26-30]. Age justification: we synthetically set 49.4 Mya as the minimum age of the node and 156.3 Mya as the soft upper bound.

Calibration F. Node: Crown lineage Siluriformes. MRCA: the specimen from Bolivia, Brazil and Argentina which dated from the Late Campanian and Early Maastrichtian (73.0-68.0 Mya) first described by Gayet and Meunier in 2003[27,29,31-34]. We therefore set 68.0 Mya as the minimum age of Siluriformes and 156.3 Mya as the soft Maximum age.

Calibration G. Node: Stem lineage of Cichlidae + Adrianichthyidae. MRCA: †*Ramphexocetus volans* from Bolca, Italy[1]. Phylogenetic identification: †*Ramphexocetus volans* coud be assigned to Beloniformes based on its caudal fin, but more likely to be of Exocetoidei for bearing an elongated dentary[1,35,36]. Age justification:as the deposits with fish fossils at Bolca was bound up with the zone dated as 49.11 Mya, we applied the date as the minimum age offset[37,38]. Inferred from the probable position of Ramphexocetus, the earliest deposits containing the related fish faunas were estimated in the Barremian (130.8-126.3 Mya). 130.8 Mya then derived as the soft maximum bound [1,18,39-41].

Supplementary Figure S1. Number comparison between raw reads and mapped reads among 14 species.

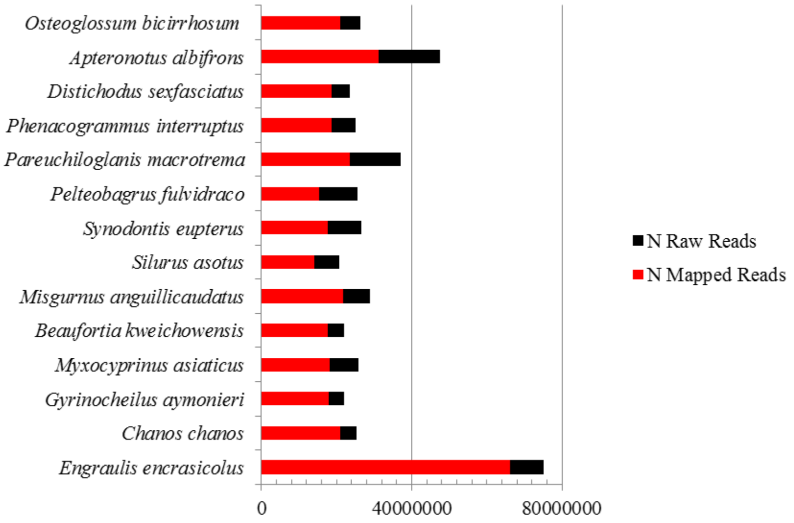

Supplementary Figure S2. Length distribution (100 bp) of mapped reads in14 species.

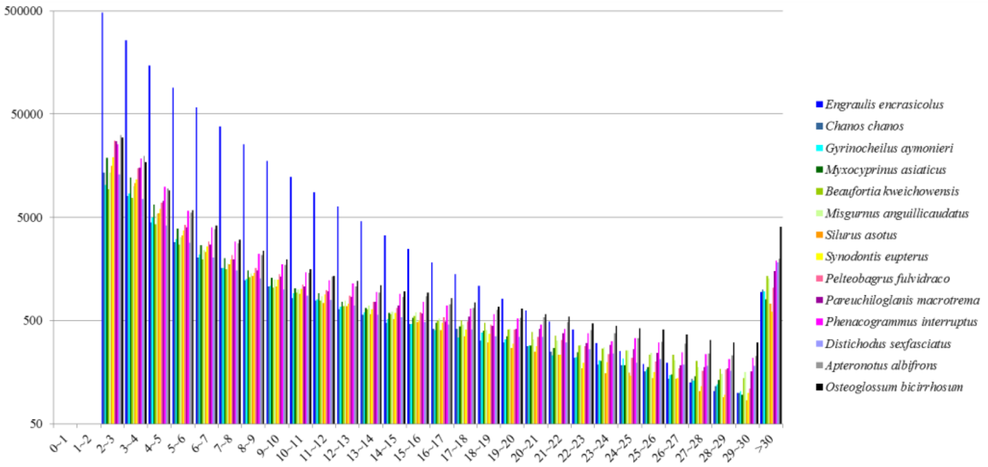

Supplementary Figure S3. Density distribution of lengths (100 bp) in contigs assembled from data of 14 species.

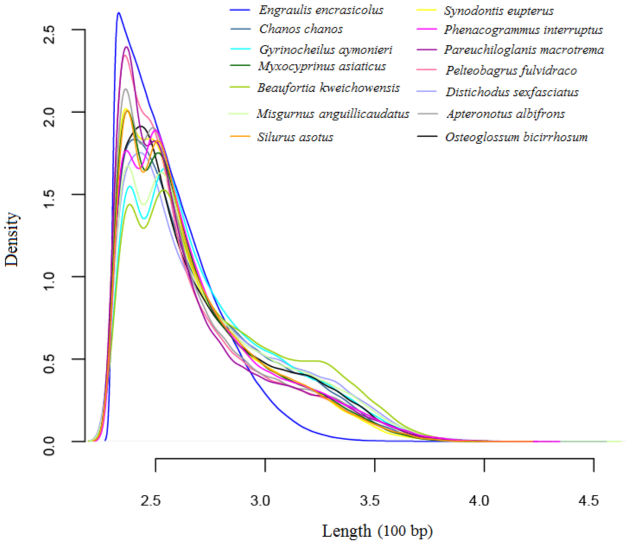

Supplementary Figure S4. Number comparison between orthologous genes and contigs from 14 species data.

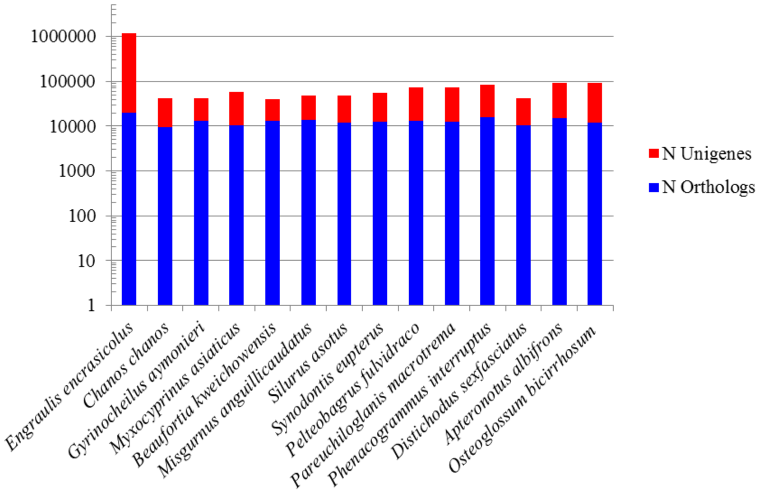

Supplementary Figure S5. Orthologous genes length distribution before and after trimming over 14 species data.

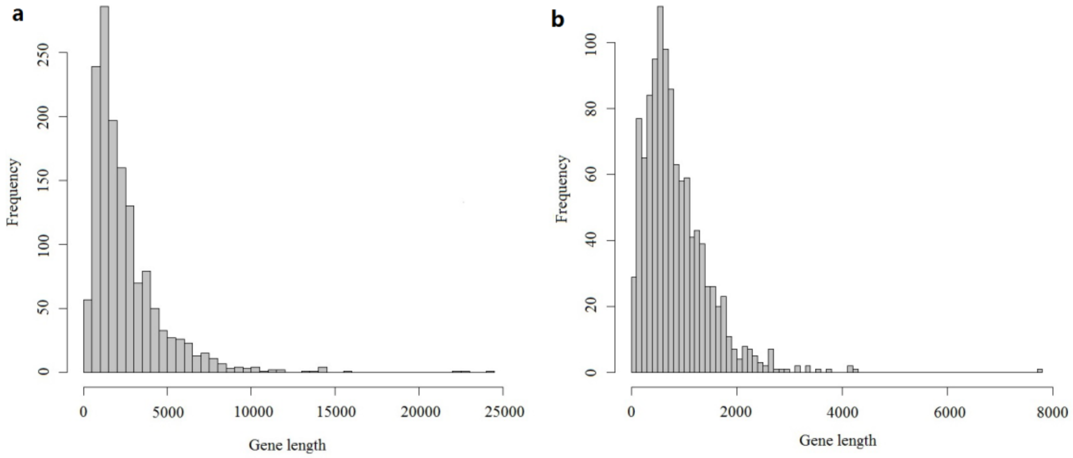

Supplementary Figure S6. Ortholog genes occupancy representation of all 22 species.

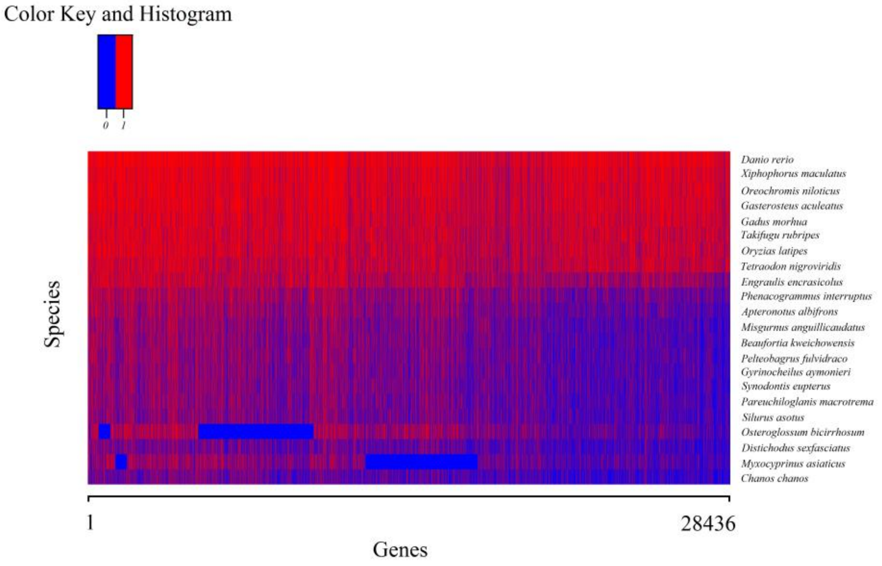

Supplementary Figure S7. Density plots of parameters in the tests for long-branch attraction and saturation on 129 genes.

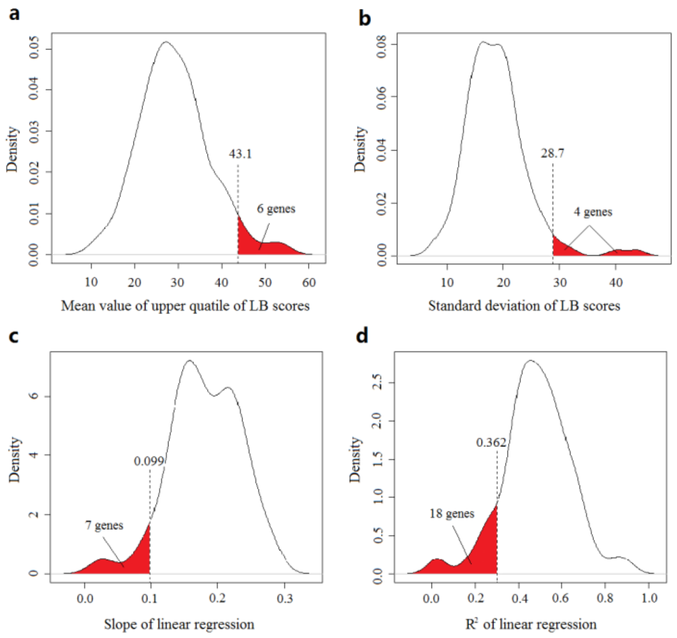

Supplementary Figure S8. Best scoring maximum-likelihood tree of Otocephala from 18 actinopterygians derived from the concatenated nuclear matrix of 1110 genes without gaps (84,201 positions, unpartitioned) implemented in in RAxML under GTRGAMMAI model.

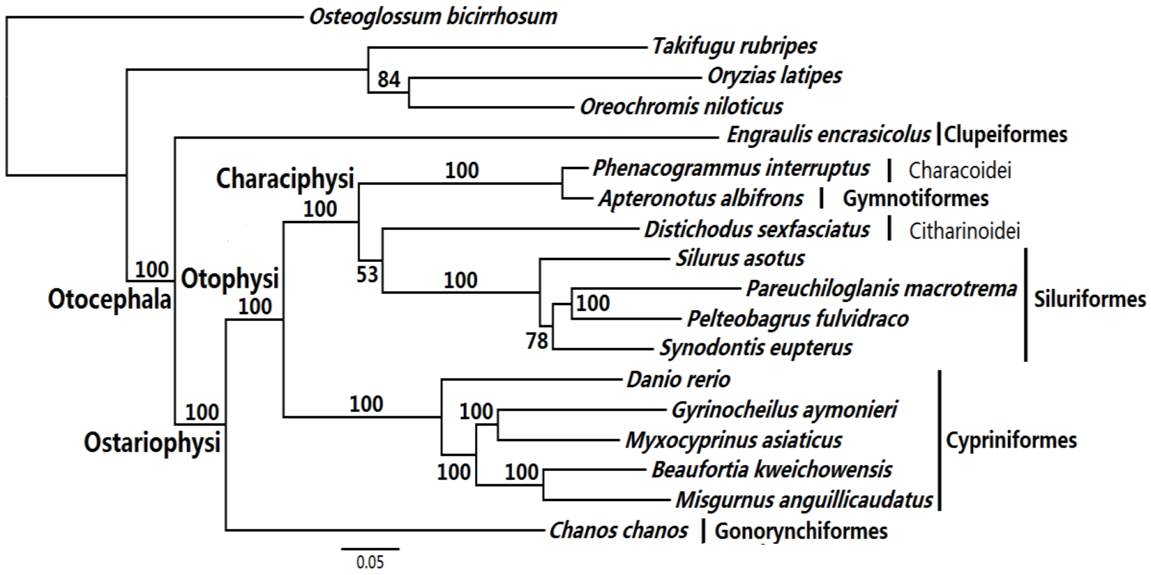

Supplementary Figure S9. The best-scoring maximum-likelihood tree of Otocephala from 18 actinopterygians based on the concatenated nuclear matrix of the 1110 genes with half gaps (623,510 positions, excluding the third codon positions and unpartitioned) implemented in in RAxML under GTRGAMMA model.

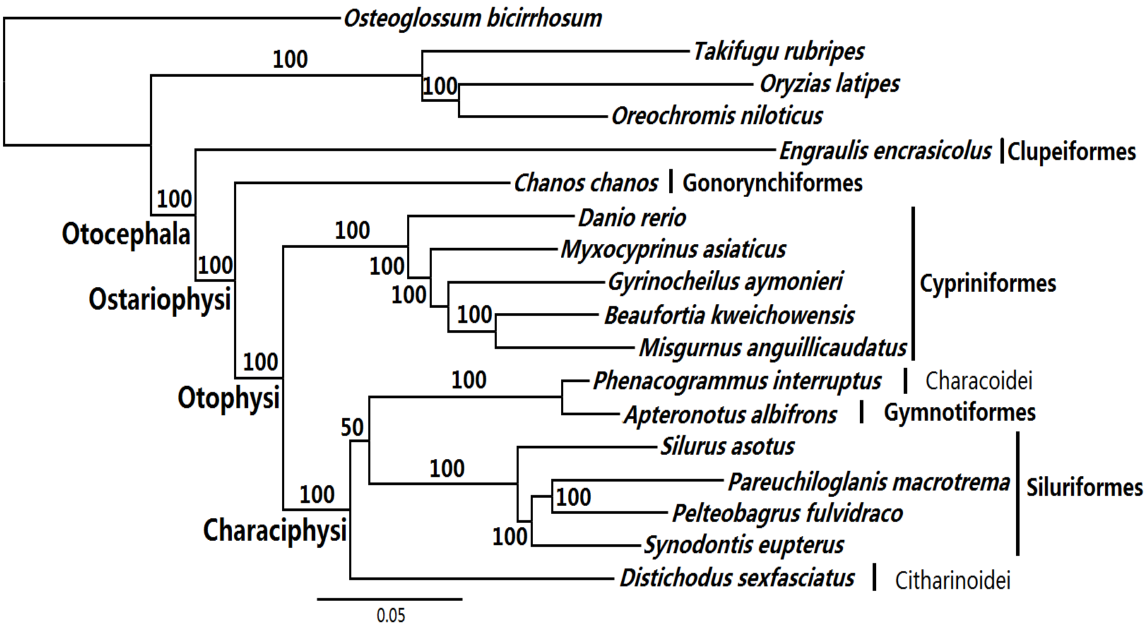

Supplementary Figure S10. The best-scoring maximum-likelihood tree of Otocephala from 18 actinopterygians based on the concatenated nuclear matrix of the 1110 genes with half gaps (935,265 positions) implemented in in RAxML under GTRGAMMA model and partitioned by genes.

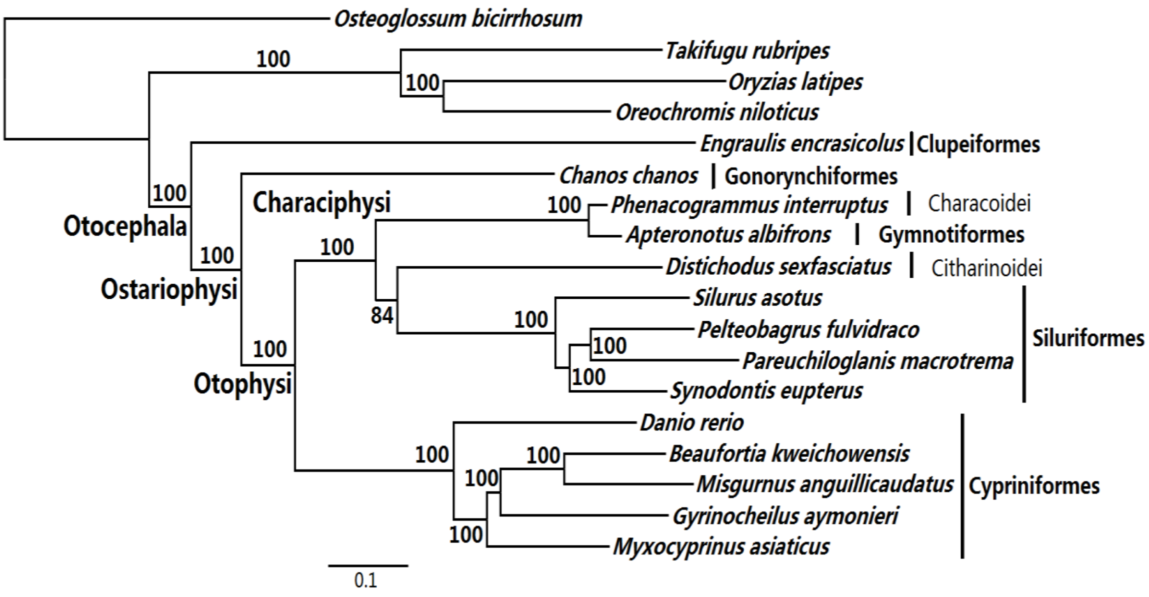

Supplementary Figure S11. The best-scoring maximum-likelihood tree of Otocephala from 18 actinopterygians based on the concatenated protein matrix of the 1110 genes without gaps (28,067 positions, unpartitioned) implemented in RAxML under PROTGAMMAJTTF model.

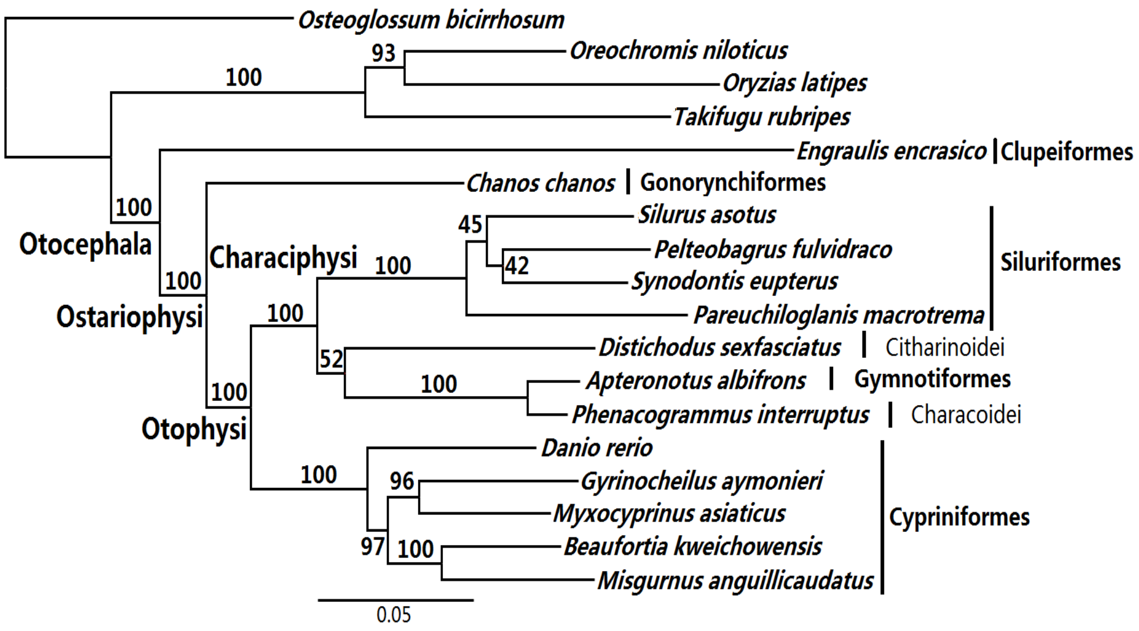

Supplementary Table 1. Time estimation (Mya) comparisons of Otocephala for basal nodes between the present and previous studies. MRCA indicates the most recent common ancestor for the lineage. Numbers represent the means and ranges of 95% posterior probabilities of lower and upper values, respectively.

**Node (MRCA)**	**This study**	**Chen et al. (2013)**	**Near et al. (2012)**	**Nakatani et al. (2011)**	**Santini et al. (2009)**	**Peng et al. (2006)**	**Saitoh et al. (2011)**
**Otocephala**	176(193-160)	177		265(286-243)	151(153-149)	279(293-264)	251(273-230)
**Ostariophysi**	153(164-137)	164	210(228-193)	261(282-240)	128(134-125)		239(260-218)
**Gonorynchiformes**	153(164-137)		183(202-165)	254(276-232)		242	228(250-206)
**Otophysi**	133(145-121)	153(181-146)	171(188-153)	248(268-227)		251	220(240-198)
**Cypriniformes**	91(99-85)	118(140-95)	95(113-79)	160(186-130)	92(123-56)	183	156(176-136)
**Characiphysi**	106(119-97)	136(153-121)		226(245-206)		210	185(207-164)
**Gymnotiformes**	29(34-26)	71(98-46)	71(87-56)	189(212-166)		150	
Characoidei	29(34-26)	93(109-78)	99(120-79)	220(240-201)	80(84-68)	203	160(183-139)
Citharinoidei	96(110-86)	79(115-40)					
**Siluriformes**	83(86-74)	79(86-72)	106(123-90)	180(198-162)	88(98-77)	173	125(148-104)

Supplementary Table 2. Samples and statistical information of data for analysis in this study. MRCA indicates the most recent common ancestor for the lineage. Numbers represents the means and ranges of 95% posterior probabilities of lower and upper values, respectively.

**Order/Suborder**	**Family**	**species**	**Tissue**	**Data type**	**N Raw Reads**	**N Mapped Reads**	**Discarded Reads (%)**	**Total N Contigs**	**N Bases (Mb)**	**N Contig >1000 bp**	**N50 (bp)**	**Contig Length (bp)**			**Total N Orthologs**	**Length after trimming (bp)**	**Missing Data (%)**	**Sources**	**Accession No.**
												**Max**	**Min**	**Mean**					
**Clupeiformes**	Engraulidae	*Engraulis encrasicolus*	Muscle	Genome	75,051,720	66,260,774	11.7	1,163,967	490,876,900	46,682	455	33,378	201	422	19,709	315,519	66.3	NCBI	SRR922010
**Gonorynchiformes**	Chanidae	*Chanos chanos*	Liver	Transcriptome	25,348,521	20,875,082	17.6	42,828	30,078,213	7,974	1,095	14,905	201	702	9,619	745,208	20.3	This study	SAMN04572088
**Cypriniformes**	Gyrinocheilidae	*Gyrinocheilus aymonieri*	Liver	Transcriptome	21,944,560	17,984,278	18	41,270	30,326,022	8,314	1,113	16,308	201	735	12,928	807,495	13.7	This study	SAMN04572089
	Catostomidae	*Myxocyprinus asiaticus*	Liver	Transcriptome	25,752,638	18,062,118	29.9	57,952	36,235,153	8,930	881	11,408	201	625	10,442	842,099	10	This study	SAMN04572090
	Cyprinidae	*Danio rerio*	Liver	Genome				43,153	64,757,328	24,366	2,070	83,298	3	1,514	25,550	928,107	0.8	Ensembl	GCA_000002035.3
	Gastromyzontidae	*Beaufortia kweichowensis*	Liver	Transcriptome	22,134,897	17,616,487	20.4	40,041	33,601,833	10,070	1,435	14,222	201	839	13,440	876,961	6.2	This study	SAMN04572091
	Cobitidae	*Misgurnus anguillicaudatus*	Liver	Transcriptome	28,971,984	21,842,359	24.6	49,125	36,685,847	10,158	1,212	33,142	201	747	13,656	830,230	11.2	This study	SAMN04572092
**Siluriformes**	Siluridae	*Silurus asotus*	Liver	Transcriptome	20,659,183	14,036,638	32.1	49,240	30,902,007	7,571	874	13,447	201	628	12,227	838,044	10.4	NCBI	SRR1994457
	Mochokidae	*Synodontis eupterus*	Liver	Transcriptome	26,519,792	17,623,509	33.5	56,194	33,993,789	8,288	832	13,920	201	605	12,682	857,761	8.3	This study	SAMN04572093
	Bagridae	*Pelteobagrus fulvidraco*	Liver	Transcriptome	25,588,131	15,310,524	40.1	72,239	43,226,156	10,274	857	14,270	201	598	13,318	872,101	6.8	NCBI	SRR1994459
	Sisoridae	*Pareuchiloglanis macrotrema*	Liver	Transcriptome	37,166,756	23,512,618	36.7	72,124	45,581,943	11,051	984	14,438	201	632	12,625	888,334	5	NCBI	SRR1994404
**Characiformes**																			
**Characoidei**	Alestidae	*Phenacogrammus interruptus*	Liver	Transcriptome	25,137,166	18,656,044	25.8	84,708	57,424,530	14,097	1,012	18,294	201	678	16,171	907,473	3	This study	SAMN04572094
**Citharinoidei**	Distichodontidae	*Distichodus sexfasciatus*	Liver	Transcriptome	23,557,723	18,672,609	20.7	42,879	35,135,874	9,616	1,455	15,576	201	819	10,688	827,739	11.5	This study	SAMN04572095
**Gymnotiformes**	Apteronotidae	*Apteronotus albifrons*	Liver	Transcriptome	47,555,467	31,312,721	34.2	91,219	59,892,817	14,694	1,019	14,668	201	657	14,835	878,555	6.1	This study	SAMN04572096
**Outgroups**																			
**Osteoglossiformes**	Osteoglossidae	*Osteoglossum bicirrhosum*	Mix	Transcriptome	26,267,820	20,908,166	20.4	91,839	72,520,735	18,272	1,450	27,857	201	790	11,795	891,251	4.7	This study	SAMN04572097
**Perciformes**	Cichlidae	*Oreochromis niloticus*		Genome				26,763	47,772,285	18196	2283	66,006	105	1,785	24,196	910,878	2.6	Ensembl	GCA_000188235.1
**Beloniformes**	Adrianichthyidae	*Oryzias latipes*		Genome				24,674	35,920,368	13,653	1869	78,425	66	1,456	22,577	858,069	8.3	Ensembl	
**Tetraodontiformes**	Tetraodontidae	*Takifugu rubripes*		Genome				47,841	90,855,693	35,049	2,367	65,562	60	1,899	22,742	870,777	6.9	Ensembl	

Supplementary Table 3. Genes with bias tested by four indices and their scores.

**LB indices for sequence bias test**	**ID of OG**	**Value**
Mean value of upper quatile of LB scores	18266	43.16494717
	25045	45.19813748
	11882	45.92506888
	1128	49.71070134
	28349	53.08165821
	28316	53.86428764
Standard deviation of LB score	23312	30.27471538
	28349	32.0154541
	1128	40.18805599
	28316	43.78592003
Slope of linear regression	1128	0.017497769
	25045	0.030878053
	28316	0.031728881
	19593	0.071129321
	4613	0.084183785
	4256	0.089646113
	1057	0.096337926
R^2 of linear regression	1128	0.010943442
	25045	0.026362617
	28316	0.035812414
	3731	0.166949005
	19593	0.172740166
	1140	0.232006356
	24669	0.239196836
	4256	0.241577015
	4813	0.248065359
	4613	0.255677203
	3392	0.275358602
	23703	0.275919964
	19055	0.284183315
	1186	0.291957122
	18700	0.306788395
	3864	0.320747692
	18004	0.328643065
	3394	0.330620049

Supplementary Table 4. Results of consel test of 9 alternative topologies obtained from this study. AU tests with p-value ≥0.05 are bold. Ost: *Osteoglossum bicirrhosum*; Ory: *Oryzias latipes*; Tak: *Takifugu rubripes; Ore: Oreochromis niloticus*; Eng: *Engraulis encrasicolus*; Dan: *Danio rerio*; Gyr: *Gyrinocheilus aymonieri*; Myx: *Myxocyprinus asiaticus*; Bea: *Beaufortia kweichowensis*; Mis: *Misgurnus anguillicaudatus*; Sil: *Silurus asotus*; Par: *Pareuchiloglanis macrotrema*; Pel: *Pelteobagrus fulvidraco*; Syn: *Synodontis eupterus*; Phe: *Phenacogrammus interruptus*; Apt: *Apteronotus albifrons*; Dis: *Distichodus sexfasciatus*; Cha: *Chanos chanos*.

**Trees**	**OBS**	**AU**	**NP**	**BP**	**PP**	**KH**	**SH**	**WKH**	**WSH**	**Topology**
1	-2900.2	**1**	1	1	1	1	1	1	1	(Ost,((Ory,(Tak,Ore)),(Eng,(((Dan,((Gyr,Myx),(Bea,Mis))),((Sil,((Par,Pel),Syn)),((Phe,Apt),Dis))),Cha))))
2	-19.9	**0.685**	0.68	0.678	1	0.679	0.679	0.679	0.679	(Ost,((Tak,(Ory,Ore)),(Eng,((((Phe,Apt),(Dis,(Sil,((Par,Pel),Syn)))),(Dan,((Gyr,Myx),(Bea,Mis)))),Cha))))
3	0	**0.574**	0.594	0.599	0.501	0.596	0.596	0.596	0.596	(Ost,(((Ory,Ore),Tak),(Eng,(Cha,((Dan,(Myx,(Gyr,(Bea,Mis)))),(((Phe,Apt),(Sil,(Syn,(Pel,Par)))),Dis))))))
4	0	**0.537**	0.556	0.558	0.252	0.549	0.606	0.549	0.593	(Ost,(((Ore,Ory),Tak),(Eng,(Cha,(((Phe,Apt),((Sil,((Pel,Par),Syn)),Dis)),(Dan,(((Mis,Bea),Gyr),Myx)))))))
5	0	**0.517**	0.531	0.534	0.5	0.526	0.526	0.526	0.526	(Ost,((Eng,(Cha,((Dan,((Mis,Bea),(Gyr,Myx))),(((Sil,(Pel,Syn)),Par),(Dis,(Apt,Phe)))))),((Ore,Ory),Tak)))
6	260.4	2.00E-05	5.00E-05	8.00E-05	8.00E-114	5.00E-05	5.00E-05	5.00E-05	5.00E-05	(Ost,((Eng,(Cha,((Gyr,((Dan,(Mis,Bea)),Myx)),(((Sil,((Pel,Par),Syn)),Dis),(Phe,Apt))))),((Ore,Tak),Ory)))
7	306.3	3.00E-12	6.00E-08	0	9.00E-134	0	0	0	0	((((((Dan,((Bea,Mis),(Gyr,Myx))),((Dis,(Sil,Syn,(Par,Pel))),(Apt,Phe))),Cha),Eng),((Tak,Ory),Ore)),Ost)
8	1963.7	1.00E-59	6.00E-18	0	0	0	0	0	0	(Ost,((((((Dan,Gyr),(Myx,(Mis,Bea))),Cha),((Pel,(Syn,(Sil,Par))),(Dis,(Apt,Phe)))),Eng),(Ory,(Ore,Tak))))
9	2902	1.00E-38	1.00E-15	0	0	0	0	0	0	(Ost,(((Cha,((((Sil,Syn),(Pel,Par)),((Phe,Apt),Dis)),((((Gyr,Bea),Mis),Myx),Dan))),Eng),((Ore,Ory),Tak)))

1 Benton, M. J. *et al.* Constraints on the timescale of animal evolutionary history. *Palaeontol Electron* **18** (2015).

2 Arratia, G. Basal teleosts and teleostean phylogeny. *Palaeo Ichthyologica* **7**, 1-168 (1997).

3 Ogg, J. G., Hinov, L. A. & Huang, C. Jurassic. *The Geologic Time Scale 2012. Elsevier, Amsterdam*, 731-791 (2012).

4 Tintori, A. The Actinopterygian Fish Prohalecites from the Triassic of Northern Italy. *Palaeontology* **33**, 155-174 (1990).

5 Arratia, G. & Tintori, A. The caudal skeleton of the Triassic actinopterygian† Prohalecites and its phylogenetic position. *Mesozoic fishes* **2**, 129-142 (1999).

6 Arratia, G. Morphology, Taxonomy, and Phylogeny of Triassic Pholidophorid Fishes (Actinopterygii, Teleostei). *J Vertebr Paleontol* **33**, 1-138 (2013).

7 Arratia, G. Complexities of Early Teleostei and the Evolution of Particular Morphological Structures through Time. *Copeia* **103**, 999-1025 (2015).

8 Arratia, G. The monophyly of Teleostei and stemgroup teleosts. *Consensus and disagreements*, 265-334 (2001).

9 Diogo, R. Origin, Evolution and Homologies of the Weberian Apparatus: A New Insight. *Int J Morphol* **27**, 333-354 (2009).

10 Broughton, R. E., Betancur, R. R., Li, C., Arratia, G. & Ortí, G. Multi-locus phylogenetic analysis reveals the pattern and tempo of bony fish evolution. *PLoS Curr* **5** (2013).

11 Griffith, J. The Upper Triassic fishes from Polzberg bei Lunz. *Zoological Journal of the Linnean Society* **60**, 1-93 (1977).

12 Ogg, J. G. Triassic. 681-730 (2012).

13 Wenz, S. Rubiesichthys gregalis ng, n. sp., Pisces Gonorhynchiformes, du Crétacé inférieur du Montsech (Province de Lérida, Espagne). *Bulletin du Muséum national d'histoire naturelle* **6**, 275-285 (1984).

14 Poyato-Ariza, F. J. The phylogenetic relationships of Rubiesichthys gregalis and Gordichthys conquensis (Ostariophysi, Chanidae), from the Early Cretaceous of Spain. *Mesozoic fishes–systematics and paleoecology*, 329-348 (1996).

15 Brenner, P. & Geldmacher, W. Ostrakoden und Alter der Plattenkalken von Rúbies (Serria del Monsech, Prov. Lérida, NE-Spanien). *Neues Jahrbuch für Geologie und Paläontologie* **8**, 513-524 (1974).

16 Schroeder, R., Altenbach, A., Brinnel, P., Cherchi, A. & Wallrabe, H.-J. El Barremienense marine de la Serria del Montsec (Provincia de LéridaaHuesca). *Cuadernos de Geología Ibérica* **8**, 915-933 (1982).

17 Martín-Closas, C. & López-Morón, N. The charophyte fauna. *Martínez-Delclós, X. (ed.)*, 29-31 (1995).

18 Ogg, J. G., Hinov, L. A. & Huang, C. Cretaceous. *The Geologic Time Scale 2012. Elsevier, Amsterdam*, 793-853 (2012).

19 Fara, E., Gayet, M. & Taverne, L. The fossil record of Gonorynchiformes. *Gonorynchiformes and Ostariophysan Relationships: a Comprehensive Review*, 173-226 (2010).

20 Davis, M. P., Arratia, G. & Kaiser, T. M. The first fossil shellear and its implications for the evolution and divergence of the Kneriidae. *Mesozoic Fishes 5 – Global Diversity and Evolution*, 325-362 (2013).

21 Silva Santos, R. Leptolepis diasii, novo peixe fóssil da Serra do Araripe, Brasil. Boletim da Divisão de Geologia e Mineralogia do Departamento Nacional de Produção Mineral, Notas Preliminares. *Brazil: Departamento Nacional de Produção Mineral* **108** (1958).

22 Filleul, A. & Maisey, J. G. Redescription of Santanichthys diasii (Otophysi, characiformes) from the Albian of the Santana formation and comments on its implications for otophysan relationships. *Am Mus Novit*, 1-21 (2004).

23 Cope, E. D. On the Tertiary coal and fossils of Osino, Nevada. *Proceedings of the American Philosophical Society* **12**, 478-481 (1871).

24 Bruner, J. C. Comments on the Genus Amyzon (Family Catostomidae). *J Paleontol* **65**, 678-686 (1991).

25 Wilson, M. V. H. Calibration of Eocene varves at Horsefly, British Columbia, Canada, and temporal distribution of specimens of the Eocene fish Amyzon aggregatum Wilson. *Kaupia (Darmstadter Beitrage zur Naturgeschichte)* **2**, 27-38 (1993).

26 Cavender, T. M. The fossil record of the Cyprinidae. *Cyprinid fishes*, 34-54 (1991).

27 Briggs, J. C. The biogeography of otophysan fishes (Ostariophysi : Otophysi): a new appraisal. *Journal of Biogeography* **32**, 287-294 (2005).

28 Liu, J. & Chang, M. M. A new Eocene catostomid (Teleostei: Cypriniformes) from northeastern China and early divergence of Catostomidae. *Sci China Ser D* **52**, 189-202 (2009).

29 Near, T. J. *et al.* Resolution of ray-finned fish phylogeny and timing of diversification. *Proceedings of the National Academy of Sciences of the United States of America* **109**, 13698-13703 (2012).

30 Chen, W. J., Lavoue, S. & Mayden, R. L. Evolutionary origin and early biogeography of otophysan fishes (Ostariophysi: Teleostei). *Evolution; international journal of organic evolution* **67**, 2218-2239 (2013).

31 Gayet, M. & Meunier, F. J. Paleontology and palaeobiogeography of catfishes. *Catfishes* **2**, 491-522 (2003).

32 Peng, Z., He, S., Wang, J., Wang, W. & Diogo, R. Mitochondrial molecular clocks and the origin of the major otocephalan clades (Pisces: Teleostei): A new insight. *Gene* **370**, 113-124 (2006).

33 Lundberg, J. G., Sullivan, J. P., Rodiles-Hernandez, R. & Hendrickson, D. A. Discovery of African roots for the Mesoamerican Chiapas catfish, Lacantunia enigmatica, requires an ancient intercontinental passage. *P Acad Nat Sci Phila* **156**, 39-53 (2007).

34 Betancur-R, R. Molecular phylogenetics and evolutionary history of ariid catfishes revisited: a comprehensive sampling. *BMC evolutionary biology* **9** (2009).

35 Rosen, D. E. & Parenti, L. R. Relationships of Oryzias and the groups of atherinomorph fishes. *Am Mus Novit* **2719**, 25 (1981).

36 Lovejoy, N. R., Iranpour, M. & Collette, B. B. Phylogeny and jaw ontogeny of beloniform fishes. *Integr Comp Biol* **44**, 366-377 (2004).

37 Medizza, F. Il nannoplancton calcareo della Pesciara di Bolca (Monti Lessini). *Studi e Ricerche sui Giacimenti Terziari di Bolca* **2**, 433-444 (1975).
38 Anthonissen, D. E. & Ogg, J. G. Appendix 3: Cenozoic and Cretaceous biochronology of planktonic foraminifera and calcareous nannofossils. *The Geologic Time Scale 2012*, 1083-1127 (2012).
39 Forey, P. L. & Longbottom, A. Bony fishes. *Fossils of the Gault Clay*, 261-296 (2010).
40 Gale, A. S. & Owen, H. G. Introduction to the Gault. *Fossils of the Gault Clay*, 1-15 (2010).
41 Nolf, D. Otoliths. *Fossils of the Gault Clay*, 270-274 (2010).
